# Fractures of the neck of the fifth metacarpal bone, treated by percutaneous intramedullary nailing: surgical technique, radiological and clinical results study (28 cases)

**DOI:** 10.11604/pamj.2014.18.187.3347

**Published:** 2014-07-04

**Authors:** Hassan Boussakri, Mohamad Elidrissi, Mohamad Azarkane, Soufiane Bensaad, Mohammed Bachiri, Mohamed Shimi, Abdelhalim Elibrahimi, Abdelmajid Elmrini

**Affiliations:** 1 Department of Orthopaedic Surgery B4, CHU Hassan II Hospital, University of Sidi Mohammed Ben Abdellah, Fez, Morocco

**Keywords:** Metacarpal neck fracture, Boxer's fracture, intramedullary nailing, minimally invasive.

## Abstract

This study report the results in 28 patients affected by closed fractures of the neck of the fifth metacarpal bone (boxer's fracture), treated with percutaneous elastic intramedullary nailing using a single wire, to verify the effectiveness of this surgical treatment. We reviewed the results of 28 patients treated with A single Kirschner wire (K-wire) pre-bent in a lazy-S fashion with a mild bend at approximately 5 millimeters, The K-wire is inserted blunt end first in an antegrade manner and the fracture reduced as the wire is passed across the fracture site The wire is usually removed with pliers post-operatively at four weeks in the fracture clinic. The follow-up period averaged of 20,75 months. The parameters evaluated included angulation, rotational alignment, postoperative metacarpophalangeal (MCP) range of motion, and time to union. We opted for this treatment in all cases, regardless volar angulation of the metacarpal head, malrotation of the fifth finger and associated or/no with a severe swelling of the hand. All the patients were reviewed clinically and radiologically at an average of 20,75 months after surgery. At the final follow-up, no patient reported residual pain and All fractures proceeded to bony union but we have one fracture had to be revised for failed fixation and three superficial wound infections needed antibiotic treatment. We recommend that this minimally invasive: percutaneous intramedullary nailing using a single k-wire in all metacarpal neck fracture( boxers’ fractures), especially when severe swelling of the hand is present, with good functional results and low morbidity.

## Introduction

Fractures of the metacarpal bones are very common injuries of the skeletal system and, in approximately 50% of the cases, involve the neck of the fifth metacarpal bone [1]. The Metacarpal neck fractures are among the most common of hand fractures with those involving the fifth metacarpal (boxer's fractures) being the most common [2]. These fractures result from a longitudinal compression force acting on a flexed metacarpophalangeal joint (MCP) — usually when a clenched fist strikes a solid object, The resultant fracture is usually unstable with volar angulation due to comminution of the volar cortex and the deforming action of the interossei [3]. These fractures are frequently observed in active young men, occur in the dominant hand and are typical injuries of aggression (boxer's fractures) [4, 5].their treatment can be problematic [3] ,many treatments are numerous: functional treatment by a simple syndactylisation with risk of secondary displacement [6, 7];the Conservative treatment consists of a cast immobilization, but sometimes source of skin complications [8, 9], and there are Various fixation techniques in use are: a direct osteosynthesis [10]; percutaneous pinning [11]; plating [12] and percutaneous transverse pinning [1].

The purpose of this study was to report the medium-term results in 28 patients affected by closed fractures of the neck of the fifth metacarpal bone (boxer's fracture), treated with percutaneous elastic intramedullary nailing using a single wire, to verify the effectiveness of this surgical treatment.

## Methods

Our series include 28 Cases of fractures of the neck of the fifth metacarpal bone (boxer's fracture), surgically treated. Complex injuries or unreliable patients were ***not included*** for this treatment and ***we excluded*** from this study: the fracture of the child, articular fracture and open fracture. The rotational deformity of the fifth finger and/or a palmar angulation of the fracture was never an exclusion criterion. The Patient demographics including age, sex, occupation, handedness and other associated medical problems were collected, and the mechanism of injury was noted and the side involved was clinically examined for rotational deformity.

Radiographs measured the angulation at the fracture side; we use a goniometer to calculate the angulation. Operative data was collected regarding time to surgical intervention, Anaesthetic mode, tourniquet. Length of stay in the hospital was documented, and Post operatively patients were assessed clinically and radiologicallly.

Ranges of movements at the métacarpophalangeal (MCP) joint and inter phalangeal (IP) joint, were assessed using **TAM**(total active motion) and **TPM** ( total passive motion) as well as the presence of any rotational deformity. From the radiographic point of view, antero-posterior (AP) and latero-oblique X-rays were taken to assess the possible presence of a residual deformity of the fifth finger. All the patients were reviewed clinically and radiographically from 5 to 37 months after surgery (average 20,75 months and SD= 10,45, median 20,5).

### **Surgical technique** (Figure 1, Figure 2)

**Figure 1:**
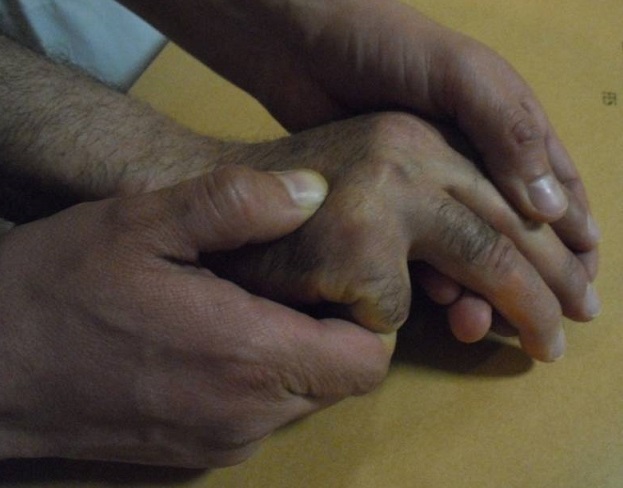
Jahss maneuver

After thorough clinical history and physical examination, standard radiographs are performed in the anterioposterior (AP); latero-oblique X-rays. Fracture angulation (whatever angulation) and /or any rotational deformity were the indications for surgical intervention.the Patients were counselled about pin site complications and care, and the necessity for removal of the pin after evidence of fracture healing. Reduction of displaced metacarpal neck fractures is best accomplished by using a technique developed by Jhass (Figure 1) [13]. The fracture is anesthetized with the hematoma block. The affected finger MCP joint and the proximal interphalangeal (PIP) joints are flexed to 90°, and the fracture is reduced by applying upward pressure on the middle phalanx and downward pressure over the dorsal apex of the fracture. Flexing of the MCP joint tightens the collateral ligaments and provides a rigid lever for reduction. The finger then can be used to control or correct the rotation. After reduction, the fracture is held via pinned to maintain reduction.

**Figure 2:**
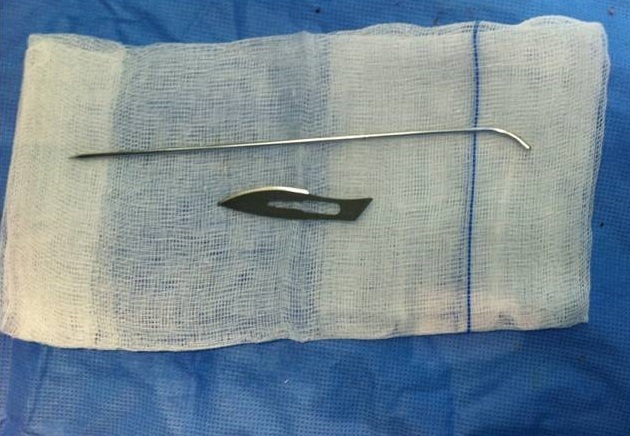
Kirschner wire (K-wire), pre-bent in a lazy-S fashion

A single Kirschner wire (K-wire) is pre-bent in a lazy-S fashion with a mild bend at approximately 5 millimeters and a longer smooth curve bent in the opposite direction (Figure 2). Depending on the metacarpal dimensions, either a 1.6 or a 2.0 millimeter (mm) K-wire is used Under image intensifier.an initial entry point is made at the base of the involved metacarpal using a 2.5 mm drill wire by hand.

A T piece mounted K-wire is then inserted blunt end first in an ante grade manner into the medullary canal after fracture reduction. Final position of the reduction is checked on the fluoroscopy and the wire is cut and a light dressing is applied and the patient is given advice about pin site care. After we realizing syndactylisation with the 4th finger.

Gentle range of movements exercises are commenced under the supervision of hand therapists. The wound and fixation are reviewed in a week to ten days. Subsequently the wire is removed around four weeks when radiological evidence of fracture healing is visualized. The wire is usually extracted post-operatively at four weeks.

## Results

### **Result** (Figure 3, Table 1, Table 2)

All the patients were reviewed clinically and radiographically from 5 to 37 months after surgery (average 20,75 months and SD = 10,45and median 20, 5). The average age of the cohort was 33,96 years (range 15-61 years and SD 13,28,median 30,5). There were 25 male and three female patients, the right hand was involved in 25 cases and the left in three, and the dominant side was injured in all but two. seventeen (17 case) of the patients were actively employed (workman in 10 case / driver in 3 case / merchant in 4 case) and unemployed in 8 case and the rest were students (3 case). The predominant mechanism of the injury in 13 patients were a punch injury. The mechanism of trauma was a direct blow in 25 patients ( in context of aggression in 5 cases and punch in 13 cases and fall in 7cases), and road traffic accident in three. In all patients, a rotational deformity of the fifth finger and/or a palmar angulation of the fracture 28 were present.

**Figure 3:**
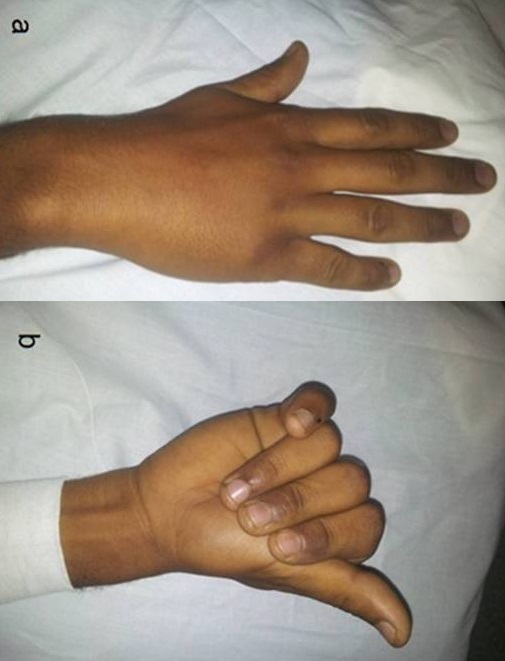
The rotational displacement of the fifth ray

**Table 1:** Characteristics of patients managed by K-wire

Patient (N)	Gender	Age (years)	Work status	Side operated	Mechanism
**1. J.T**	M	15	M(student)	D	P
**2. B. A**	M	16	M(student)	D	P
**3. Z. A**	M	18	NW	D	P
**4. B. M**	M	28	M	D	P
**5. S.Y**	M	21	NW	D	A
**6. L.A**	M	24	M	D	P
**7. C.T**	F	26	NW	D	P
**8. D.S**	M	28	M	D	P
**9. M.M**	M	30	M	D	F
**10. A. M**	M	31	M	D	P
**11. T.I**	M	36	M	D	P
**12. K. H**	M	37	M	D	P
**13. A. L**	M	41	M	D	F
**14. B. R**	M	42	M	D	A
**15. N.B**	M	43	M	D	F
**16. M. A**	M	45	M	D	RRA
**17. Y.Y**	M	48	M	D	F
**18. B.S**	F	50	M	ND	RRA
**19. M.S**	M	52	S	D	F
**20. E.L**	M	60	S	ND	F
**21. A. O**	M	41	M	D	A
**22. B.H**	M	18	M(student)	D	P
**23. A.D**	M	19	NW	D	A
**24. A.C**	M	24	M	D	P
**25. N.A**	F	28	M	D	P
**26. B. L**	M	61	S	D	RRA
**27. A.A**	M	45	M	D	A
**28. J.H**	M	24	NW	D	F

M: male; F: female; M: manual; S: sedentary; NW: not working; D: dominant; ND: non-dominant; P: punch; F: fall; RRA: road traffic accident, A: aggression

**Table 2:** Angulation before and after surgery treatment

Patient	Preoperative displacement (°)	Postoperative Displacement (°)
**1. J.T**	20°	5°
**2. B. A**	65°	3°
**3. Z. A**	60°	5°
**4. B. M**	75°	10°
**5. S.Y**	65°	5°
**6. L.A**	45°	5°
**7. C.T**	45°	5°
**8. D.S**	30°	3°
**9. M.M**	80°	10°
**10. A. M**	45	5°
**11. T.I**	30°	5°
**12. K. H**	45°	5°
**13. A. L**	50°	15°
**14. B. R**	85°	20°
**15. N.B**	45°	10°
**16. M. A**	35°	15°
**17. Y.Y**	65°	20°
**18. B.S**	35°	15°
**19. M.S**	80°	35°
**20. E.L**	50°	15°
**21. A. O**	60°	10°
**22. B.H**	60°	5°
**23. A.D**	90°	5°
**24. A.C**	60°	10°
**25. N.A**	45°	10°
**26. B. L**	45°	15°
**27. A.A**	60°	15°
**28. J.H**	65°	20°

Rotational displacement of the fifth ray was evaluated clinically: malrotation of the fifth metacarpal was diagnosed when the fifth finger was not oriented towards the scaphoid tubercle in flexion, and if the little finger was not parallel to the other fingers in extension (Figure 3). The palmar angulation of the metacarpal head was measured on the latero-oblique X-rays with a goniometer (Table 2); it measured from 20 to 90, with an average of 54,64 and SD:17,68.

The time to surgical intervention from the injury date was a mean 2,6 days (range 1 to 10 days, SD 2,46 and median 1). The procedures were performed under short general anaesthetic by a Consultant surgeon or a trainee surgeon under supervision. The mean tourniquet time was25 minutes (range 15-40 minutes).

**Figure 4:**
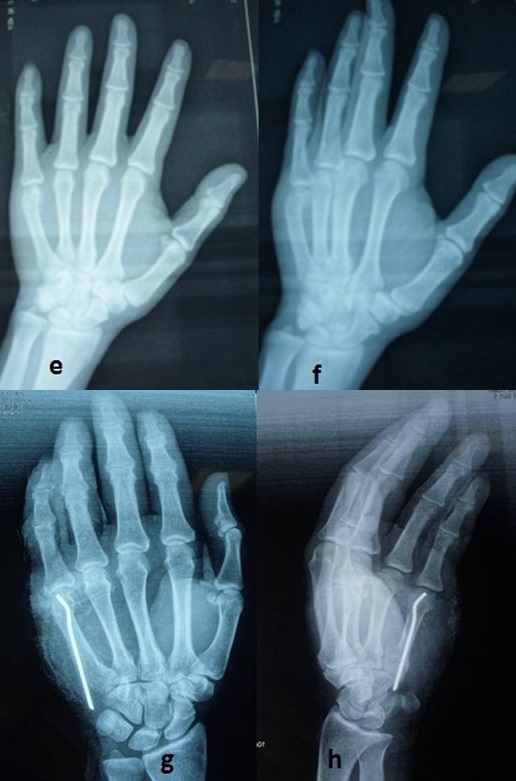
(e,f): Radiograph of a 28 years old male patient with displaced little finger metacarpal neck fracture; (g,h): Postoperative radiograph of the same patient treated with intramedullary nailing

**Figure 5:**
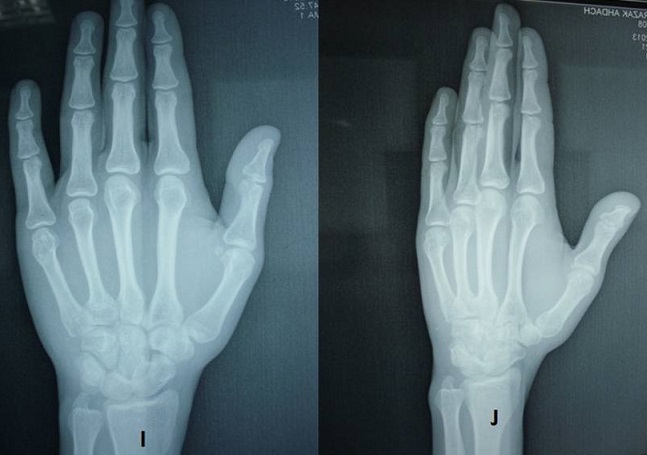
Radiological evidence of satisfactory outcome after removal of the wire of the same patient as show in Figure 4

**Anatomical results (Figure 4, Figure 5**): All fractures proceeded to radiological bony union without rotational or severe angulation deformities (Figure 4, Figure 5). The wire was extracted in all patients at a mean period of 4 weeks (range four to six weeks). All fractures were consolidated to 30 days evolution. The flip volar was initially 54,64° (20 -90°, SD = 17,68°, Median: 55). After reduction it was 10,21° (3-35°, SD = 7.3°, Median:7,5).

**Figure 6:**
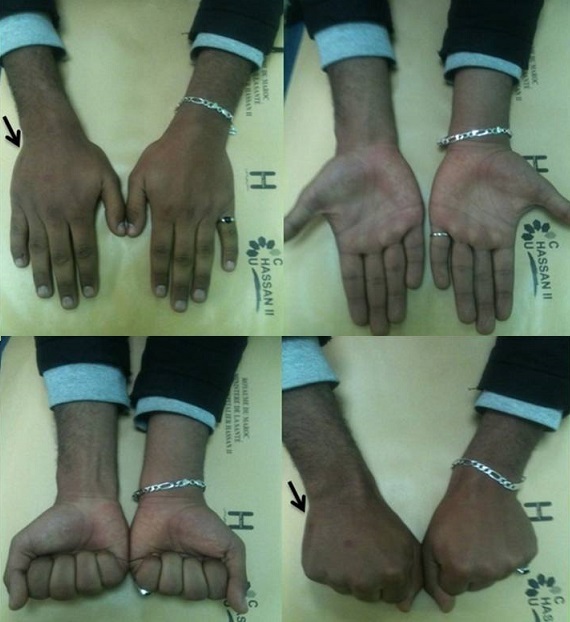
Examination shows perfect healing of the fracture without malrotation or volar angulation of the metacarpal head and a good functional results at 1 year followed-up of the same patient as show in Figure 4, Figure 5

**Functionnel results** (Figure 6): At final follow-up, the mean total passive motion was 285° (range 200°-325° SD = 23) and the mean total active motion (TAM) was 270° (range 190°-310° SD = 21). All of the patients achieved full extension of the little finger.

**Subjectif results:** 97% patients were cosmetically and functionally satisfied with the results of their surgery.

**Socioeconomic results:** Number of days off work was with 50(25-60), there is no note or partial permanent incapacity and all purely patients return to work before accident. There is only one patient presented a bad result overall TPM =250 and TMA= 170, of a complicated reflex sympathetic dystrophy.

## Discussion

The aim of this retrospective study was to assess the clinical and radiographic results of percutaneous intramedullary nailing fixation with single K-wires in fractures of the neck of the fifth metacarpal bone.the Boxer's fractures are very common injuries^13^, [14]. Which can cause impairment of hand function [15–17], When they heal in malrotation and/or in volar angulation of the metacarpal head and the result may be a loss of the grip strength and an extension deficit of the little finger.

The epidemiological study of our series is in agreement existing with other studies [18, 19]. The punch is by far the most common etiology but this concerns only rarely fracture the real boxer [20], but rather a young population impulsive and hence the preponderance of male [2]. The frequency of this etiology is certainly underestimated in our study, where the patients attach their fracture at accident the work.

The management of boxer's fracture is still Controversial [1],Various operative techniques have been proposed for the surgical treatment of these fractures [4, 21, 22].the principles of treatment include restoration of articular anatomy, stable fixation of fractures, elimination of angular or rotational deformity and rapid restoration of mobility and function [23]. Although Flexible intramedullary nailing is indicated for any displaced or unstable fracture of the metacarpal neck [24].first described by Foucher In 1975, introduced the “bouquet” technique of closed anterograde nailing of metacarpal fractures using multiple small pre-bent K-wires, is currently one of the surgical procedures of choice [11].

We report the results obtained in 28 patients with a fracture of the neck of the fifth metacarpal, treated surgically using single Kirschner wire (K-wire) and followed-up at least 20, 75 months after surgery. The majority of our patients, at surgery, presented a severe swelling of the surrounding soft tissues of the hand. We believe that the presence of a severe swelling of the soft tissues of the hand is one more reason to choose this type of treatment which minimizes the surgical trauma.

Intramedullary pinning and internal fixation with locking plates are the other two common methods of treatment for boxer's fracture. Recently, in a comparative study on fifth metacarpal neck fracture fixation, Facca et Al [22] reported better results in the series stabilized with intramedullary K-wire in comparison to a second series treated with locking plates and immediate mobilization, In this second series treated with locking plates, the authors reported a deficit, despite immediate mobilization, of the MP joint due to the adherences of the extensor. Winter et al [25] In their short-term retrospective study, with a mean follow-up of 2.7 months, reported that in the boxer's fracture intramedullary pinning gave better functional outcomes than transverse pinning, although they concluded that intramedullary pinning is technically more demanding than transverse pinning and the surgeon has a more definite learning curve [25].

To the best of our knowledge a rare documents were published on percutaneous intramedullary nailing technique – by single k-wire-for treatment of fractures of the neck of the fifth the metacarpals [21, 23]. Mohammed and Al reported The general outcome was a good hand function in a series of 15 metacarpal neck fractures and 5 diaphyseal treated by Percutaneous elastic intramedullary nailing with A single K-wire, In their retrospective study (from November 2007to august 2009).

We describe a surgical technique that contains of variety of technical of Foucher using a single wire of adequate diameter which is pre-bent to act as an elastic support With the elastic pre-bent wire with syndactylisation,adequate stability is achieved to commence early mobilization. With minimal soft tissue dissection, avoidance of periosteal stripping and flexible fixation as opposed to rigid fixation, abundant periosteal callus is generated encouraging fracture healing [11]. In addition this procedure is relatively simple, with reduced operating times, minimal radiation exposure and can be performed as day case surgery thereby reducing hospital costs [26]. The disadvantages of this technique are lack of absolute stability, wire migration, impalement of soft tissues, pin site problems, infection and the necessity for implant removal [11, 27]. In our series, At follow-up, no patient had any clinically detectable rotational deformity of the fifth finger with a consequent deficit of the grip strength and at the final radiographic examination we never observed nonunion, avascular necrosis of the fifth metacarpal head,or abnormalities of the MP joint. But there are three superficial wound infections with surrounding cellulitis needed antibiotic treatment, removal of wire (at three weeks).

**Figure 7:**
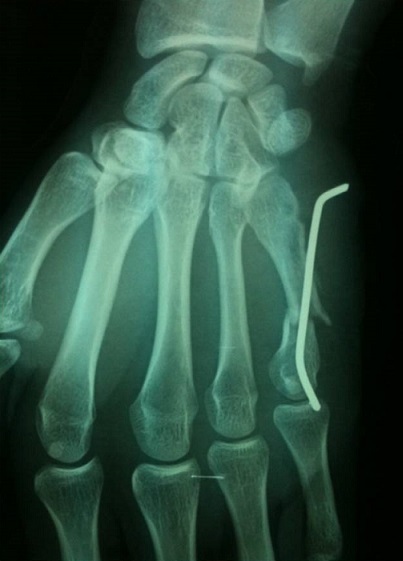
One case as the wire had backed out loosing the reduction at the fracture site

We had five patients had buried wires and the wire removed in the operatory block. It is worth mentioning that the metacarpal must be perforated laterally so that the extensor mechanism is not impaled by the wire. The diameter of the wire chosen depends on the bone and should be strong enough to resist minimal forces during early mobilization. Foucher's bouquet osteosynthesis method was described using three 0.8 mm wires. We aim to leave the wire protruding from the skin for ease of removal in the outpatient clinic, the wire is cut flush with the bone and is buried to allow wound closure. Wire migration has been reported to be common with this method and Foucher recommends leaving sufficient length of the wire to allow easy secondary removal. We had to revise one fracture as the wire had backed out loosing the reduction at the fracture site (Figure 7); in these cases it is imperative to get ‘hockey stick’ bend in the K wire at the correct length to be able to adequately hold the smaller distal fragment. All patients in our study except the one requiring revision were generally satisfied about the surgical experience.

Our study has demerits in that few patient numbers are involved and that it is a retrospective analysis, however we have highlighted the merits of a very simple technique that saves operative time, adequately stabilizes the metacarpal neck fracture, promotes early mobilization, fewer complication rates and in general obtains a satisfactory outcome in the majority of patients. Larger, prospective studies may be required to validate the technique.

## Conclusion

In conclusion, we recommend that this minimally invasive: percutaneous intramedullary nailing using a single in all boxers’ fractures, especially when severe swelling of the hand is present, with good functional results and low morbidity.
